# Early Detection of Simian Immunodeficiency Virus in the Central Nervous System Following Oral Administration to Rhesus Macaques

**DOI:** 10.3389/fimmu.2013.00236

**Published:** 2013-08-14

**Authors:** Jeffrey M. Milush, Hui-Ling Chen, Ginger Atteberry, Donald L. Sodora

**Affiliations:** ^1^Department of Medicine, Division of Experimental Medicine, University of California San Francisco, San Francisco, CA, USA; ^2^Seattle Biomedical Research Institute, Seattle, WA, USA; ^3^Southwestern Medical Center, University of Texas, Dallas, TX, USA

**Keywords:** SIV, oral transmission, CNS, acute infection, rhesus macaque, monkey model, HIV

## Abstract

The timing of HIV dissemination to the central nervous system (CNS) has the potential to have important implications regarding HIV disease progression and treatment. The earlier HIV enters the CNS the more difficult it might be to remove with antiretroviral therapy. Alternatively, HIV may only enter the CNS later in the course of disease as a result of disruption of the blood-brain-barrier. We utilized the simian immunodeficiency virus (SIV) infection of rhesus macaques to evaluate the oral route of infection and the subsequent spread of SIV to the CNS during the acute infection phase. A high dose oral SIV challenge was utilized to ensure a successful infection and permit the evaluation of CNS spread during the first 1–14 days post-infection. Ultrasensitive nested PCR was used to detect SIV gag DNA in the brains of macaques at 1–2 days post-infection and identified SIV gag DNA in the brain tissues from three of four macaques. This SIV DNA was also present following perfusion of the macaque brains, providing evidence that it was not residing in the circulating blood but in the brain tissue itself. The diversity of the viral envelope V1–V2 region at early times post-infection indicated that the brain viral variants were similar to variants obtained from lymph nodes. This genetic similarity between SIV obtained from lymphoid and brain tissues suggests that the founder population of viral species entered and subsequently spread without any evidence of brain-specific SIV selection. The relatively rapid appearance of SIV within the CNS tissue following oral transmission may also occur during HIV transmission where it may impact disease course as well as representing a challenge for long-term therapies and future viral eradication modalities.

## Introduction

HIV-associated neurologic disorders generally do not manifest themselves until onset of AIDS when the clinical disease can be readily observed (1). Recently, insights into HIV-induced central nervous system (CNS) abnormalities have been revealed utilizing magnetic resonance imaging (MRI). Functional MRI studies have determined that structural changes in the brains of HIV-infected persons are similar to the effects of aging in HIV-negative people (2, 3). Furthermore, alterations in brain metabolites in patients exhibiting AIDS dementia complex (ADC) have identified a decrease in the ratio of acetylaspartate to creatine in both the gray and white matter (4–6). Changes could also be observed in HIV-infected patients without ADC including an increase in the ratio of myo-inositol to creatine in white matter (2, 5, 6). Therefore, although the clinical manifestations of ADC occur during the onset of AIDS, evidence suggests that the virus has been present and inducing changes in the CNS prior to end stage disease signs (7, 8). Indeed, data from a limited number of patients indicates that HIV infects the CNS at early times post-infection (9, 10). While these studies are not conclusive, they indicate that HIV has the potential to invade the CNS prior to end stage disease although the precise timing of the HIV infection into the CNS is not known.

The ability to undertake careful and controlled studies utilizing the Rhesus macaque simian immunodeficiency virus (SIV) infection model has enabled the evaluation of CNS infection events that cannot be addressed in HIV-infected patients. Indeed, SIV has been particularly invaluable for investigating the earliest post-infection CNS events (11). Four-to-seven days following intravenous SIV infection, virus can be found within CNS tissues (11) however CNS penetrance at very early times following mucosal transmission (i.e., oral, vaginal, or rectal) have not been assessed. Viral DNA can generally be detected throughout the disease course, although viral RNA is most easily isolated at only the earliest times post-infection (prior to day 14) suggesting that viral replication is reduced as the immune response is initiated (12). The interferon response is induced early in the brain following SIV infection (13, 14) and elevated interferon beta expression can be detected as early as 4 days following an intravenous inoculation of SIV (13). A concomitant induction of interleukin 10 (IL-10), tumor necrosis factor alpha, and CCL2 expression were also observed (13). Interestingly, the peak in antiviral response in the brain appears to be approximately 7 days post-intravenous inoculation, and there is some evidence that the viral RNA transcription appears to be somewhat controlled (13) and then reactivates during the chronic times post-infection (13). Finally, despite the effective control of plasma viremia by current antiretroviral drug regimens, the CNS represents a site where residual virus may be maintained resulting in acute or sub-acute neurological manifestations (15, 16).

A long-standing question in the field of neuroAIDS is the source of the HIV found in the cerebral spinal fluid (CSF) during chronic infection. The variants replicating at later times post-intravenous infection appear to be related to these early species suggesting an activation of latent virus (12, 17). Some studies analyzing CNS viral variants following intravenous inoculation have identified viral variants that appear to be specific to the brain (18) whereas others did not find evidence for any variants that were particularly brain-tropic (19, 20). One source of these different findings may be the result of a mixing of the virus produced in the CNS with virus entering from the peripheral blood circulation (21). Using highly sensitive nested PCR, we previously observed that SIV rapidly spreads throughout the systemic lymphoid system within 1–7 days post-oral mucosal transmission (22). Here we investigate the ability of SIV to spread to the brain in these same macaques following oral mucosal SIV inoculation and evaluate genetic differences of SIV variants in the CNS versus the peripheral tissues at these earliest time points. Our findings support a model of rapid SIV spread throughout the body, including the brain, during the earliest times post-infection. Furthermore, the viral variants detected in the brain were similar to those found in the peripheral tissues suggesting that the viral species that cross the mucosal surface become equally distributed throughout the body, including in CNS tissues.

## Materials and Methods

### Ethics statement

All animals used in this study were housed at the California National Primate Research Center (CNPRC) in accordance with the recommendations of the Association for Assessment and Accreditation of Laboratory Animal Care International Standards and with the recommendations in the Guide for the Care and Use of Laboratory Animals of the National Institutes of Health. These animal studies were approved by the Institutional Animal Use and Care Committee at the University of California, Davis, in accordance with NIH guidelines and the recommendations set forth by the Weatherall Report. Animal housing and care was conducted according to the Guide for the Care and Use of Laboratory Animals and the United States Department of Agriculture Animal Welfare Act. Animals were housed in an air-conditioned facility with an ambient temperature of 21–25°C and a 12-h light/dark cycle. Each animal was individually housed during the study period in suspended stainless steel wire-bottom cages and with commercial primate diet, fresh fruit once daily, and freely available water. All of the animals in our studies were terminated at specific time points before progressive disease developed. At predetermined time points (i.e., 1, 2, 4, 7, and 14 days post-inoculation), the animals were humanely euthanized using sodium pentobarbital overdose in accordance with CNPRC and Federal guidelines.

### Animal inoculations and virus stock

A total of 12 animals used in this study were colony-bred rhesus macaques (*Macaca mulatta*) housed at the CNPRC. Neonatal macaques ranged from 3 to 15 days of age. Juvenile macaques ranged from 1.75 to 3.3 years of age. Each macaque was orally inoculated with two doses of SIVmac251-5/98 each with a 50% tissue culture infectious doses (TCID50) of 1 × 10^5^ (23, 24) to ensure infection and was administered under ketamine hydrochloride anesthesia (10 mg/kg) by methods previously described (25). At the predetermined times post-oral inoculation (i.e., 1, 2, 4, 7, and 14 days post-inoculation), the macaques were humanely euthanized and numerous lymphatic and non-lymphatic tissues collected. These analyses were undertaken in both neonate and juvenile macaques and no distinction in the rate of viral spread was observed with respect to age. The analysis of non-brain tissues for the presence of SIV following the oral SIV infection of these same macaques has been previously described in detail (22).

### Tissue collection and sample processing

At necropsy, lymphoid and brain tissues (i.e., cerebrum and cerebellum) were harvested and snap frozen in liquid nitrogen and stored at −80°C for DNA or RNA isolation. The 2, 4, 7, and 14 days as well as 1 day infected macaques 33711 and 34262 were not perfused prior to tissue collection. One day infected macaque 33098 underwent perfusion of the brain with sterile 1× phosphate buffered saline (PBS) to eliminate peripheral blood contamination. Perfusion was performed while the animal was under deep ketamine hydrochloride anesthesia. The brain was perfused by cannulating the left ventricle, clamping the descending aorta and vena cava, and cutting the right atrium to allow for escape of the PBS and peripheral blood. A total of 1L of PBS was slowly pushed through the system by hand in 60 ml increments. The 1 day infected macaque 33202 underwent a whole body perfusion prior to tissue collection by the same technique described above, however, the descending aorta and vena cava were not clamped. Tissues were preserved by three methods. Samples were snap frozen in liquid nitrogen and stored at −80°C for DNA and RNA isolation. Tissues were also fixed in Streck’s tissue fixative buffer (Streck Laboratories, Inc., Omaha, NE, USA) or 10% neutral-buffered formalin prior to being paraffin embedded.

### Assessment of SIV *gag* DNA by nested PCR

Genomic DNA isolation and nested PCR were performed as previously described (22). The nested PCR approach provided the sensitivity to repeatedly detect one to five copies of SIV plasmid DNA. Each DNA sample was tested in 3–20 replicates. Human and SIV-negative macaque PBMC were used as negative controls. Further internal controls were established through a re-analysis of selected tissues that continued to result in a similar number of PCR positive/negative reactions.

### Heteroduplex mobility assay assessment of SIV *env* V1–V2 diversity

A 590 bp fragment encompassing the V1–V2 regions of SIV was PCR amplified in triplicate from lymphoid and brain tissues using a similar nested PCR approach with primer sets that have previously been described (23). The Env V1–V2 PCR products of the triplicate reactions were combined. Then, 20 μl of PCR product were added to 2 μl of 10× annealing buffer [1 M NaCl, 100 mM Tris (pH 7.8), 20 mM Ethylenediaminetetraacetic acid], and the samples were heated to 100°C for 5 min before rapidly cooling the reaction on ice. The product was then mixed with 4 μl of a 5× loading dye (25% Ficoll, 1% Orange G) and loaded onto a 16% non-denaturing polyacrylamide gel and electrophoresed 2 h at 250 V. The bands were visualized by ethidium bromide staining. By heating and rapidly cooling the samples, the two strands of DNA are rapidly re-annealed and form mismatches if the product is not homogenous. Mismatches cause bulges in the DNA that retard its migration in the gel.

### Sequence analysis of SIV *env* V1–V2 region

The 590 bp PCR fragment encompassing the V1–V2 regions of Env was directly sequenced using the Applied Biosystems Big Dye Terminator 3.1 chemistry and analyzed on an Applied Biosystems capillary instrument. When double peaks were observed in the sequences, the nucleotide with the largest peak at the position was inserted. SIV V1–V2 Env sequences obtained from different tissues were aligned and a phylogenetic tree using the Neighbor Joining method with 500 bootstrap values was generated by software MEGA version 4.1 (www.megasoftware.net, Center for Evolutionary Functional Genomics, Tempe, AZ, USA).

## Results

### Detection of SIV DNA in the brain post-oral inoculation

**Figure 1:**
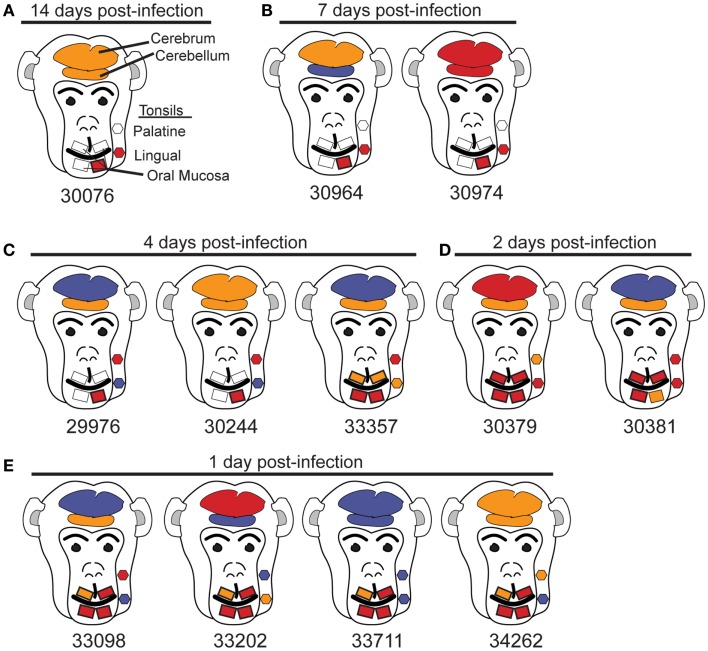
**Anatomic distribution of SIV *gag* DNA-positive tissues of the head**. **(A–E)** Tissues are depicted in their general anatomical position. **(A,B,D)** Juvenile macaques. **(C)** 29976 and 30244 are juvenile macaques and 33357 is a neonate. **(E)** Macaques 33098 and 33202 are juveniles and 33711 and 34262 are neonates. To minimize the possibility that SIV in the blood of tissues would be detected by PCR, the head of macaque 33098 and the whole body of 33202 were perfused with PBS prior to collecting tissues. Despite perfusing these animals, SIV *gag* DNA was detected in the brain. Results are based on the number of SIV gag DNA-positive reactions with a minimum of three reactions for non-brain tissues and seven reactions for cerebrum and cerebellum. Tissues are depicted as follows: red, 50% or more of the PCR reactions were positive; orange, less than 50% of PCR reactions positive; blue, no PCR reactions positive; gray, tissues not available.

Although SIV has been detected in the brain tissue by day 4–7 following intravenous SIV inoculation (11), the rate at which SIV enters the brain following mucosal transmission is not known. To address this question through an evaluation of the oral route of infection, 12 macaques were orally inoculated with the quasispecies SIVmac251 as previously described (22, 26). Assessment of non-CNS tissues of these macaques obtained at days 1, 2, 4, 7, or 14 post-oral SIV administration provided evidence that the oral cavity and upper GI tract were the likely entry points of the virus and that the virus rapidly spread to draining and peripheral lymphoid tissues (22). Here, CNS tissues (cerebrum and cerebellum) from these same 12 macaques were assessed using an ultrasensitive nested (two rounds) PCR approach (sensitivity of one to five copies of plasmid DNA (22) to detect a 597-bp SIV *gag* DNA fragment. As negative controls we included human and uninfected macaque PBMC DNA in all PCR experiments to demonstrate the specificity of our SIV *gag* PCR (previously shown (22). Previously, we observed in these 12 orally inoculated macaques that SIV could rapidly spread from the likely entry points, oral mucosa (gingival) and upper esophagus, to the blood and lymphatics within 2–4 dpi (22). Table 1 summarizes the tissues of the head and neck that were assessed for SIV *gag* DNA as well as the frequency of PCR positive results. Using the results of our nested PCR analysis, we have depicted the approximate anatomical location and color-coded the results to indicate the relative frequency of SIV *gag* PCR positive reactions in each tissue (Figure 1). Dissemination of the virus to nearly all tissues of the head and neck, including the cerebrum and cerebellum, is evident at 7 and 14 dpi by the frequent red (greater than 50% PCR positive) and orange (PCR positive but at a frequency of less than 50% of the PCR reactions) colored SIV DNA-positive tissues (Figures 1A,B). Assessment of cerebrum (larger oval in head) and cerebellum (smaller oval in head) samples at 1, 2, and 4 days post-infection indicated that the virus reached these tissues as early as 1–2 dpi (Figures 1C–E). This rate of spread following oral transmission is similar to that previously observed following intravenous inoculation (12, 13, 27) and not influenced by age as both juvenile and neonate macaques had similar frequencies of SIV DNA PCR positive reactions in the two brain tissues assessed (Table 1; Figure 1). To rule out the possibility that residual blood in brain tissues was responsible for the DNA-positive PCR reactions, we perfused two macaques (33098 and 33202) at necropsy to remove blood from the brain tissues. Despite perfusing, SIV nucleic acid was still detected in the brain tissues of these macaques indicating the virus was likely residing in cells that had migrated into the CNS tissue. However, due to the paucity of infected cells, we were unable to identify the infected cells using *in situ* hybridization (data not shown). Indeed, clusters of infected cells could not be identified until 7 dpi in lymph nodes (22). While the virus appears to be preferentially detected in the cerebellum at these early time points (seven of nine macaques assessed between 1 and 4 dpi tested positive for SIV DNA in the cerebellum compared to only four of nine testing SIV DNA-positive in the cerebrum), the results of a Fisher’s exact test were not significant (*p* = 0.3348). Importantly, SIV DNA in the blood was not detected at 1 day post-infection and was detected in less than 50% of PCR reactions performed at 2 and 4 days post-infection. In contrast to SIV DNA, detection of SIV RNA is more difficult and in our previous study of these same macaques we were unable to reliably detect SIV RNA in lymphoid tissues prior to 4–7 days post-infection (22). Similarly, attempts to amplify SIV RNA by traditional or quantitative RT-PCR from brain tissues of these 12 macaques were also unsuccessful (data not shown). These findings are the first to indicate that SIV can gain access to the CNS very early (by 1–2 dpi) following non-traumatic oral mucosal administration of virus.

**Table 1:** **Detection of SIV nucleic acid in mucosal associated tissues or lymphoid tissues**.

	J^1^	N^2^	N	J	J	J	J	N	J	J	J
	1 day	1 day	1 day	2 days	2 days	4 days	4 days	4 days	7 days	7 days	14 days
Tissues examined^3^	33098	33711	34262	30379	30381	29976	30244	33357	30964	30974	30076
Oral mucosa	+^4^	−	++	++	++	++	++	++	++	++	++
Lingual tonsil	−	−	−	++	++	−	−	+	++	++	++
Palatine tonsil	na^5^	−	−	+	++	++	++	++	na	na	na
Cerebrum	−	−	+	++	−	−	+	−	+	++	+
Cerebellum	+	−	+	+	+	+	+	+	−	++	+
PBMC post-infection	−	+	−	+	+	+	+	++	++	++	na

*^1^J, juvenile; ^2^N, neonate*.

*^3^All tissues were assessed in carefully controlled nested PCR reactions for SIV gag DNA*.

*^4^Indicates number of positive reactions to the total number of replicates tested*.

*^5^“na” indicates tissues were not assessed*.

### Systemic and CNS viral diversity assessment

**Figure 2:**
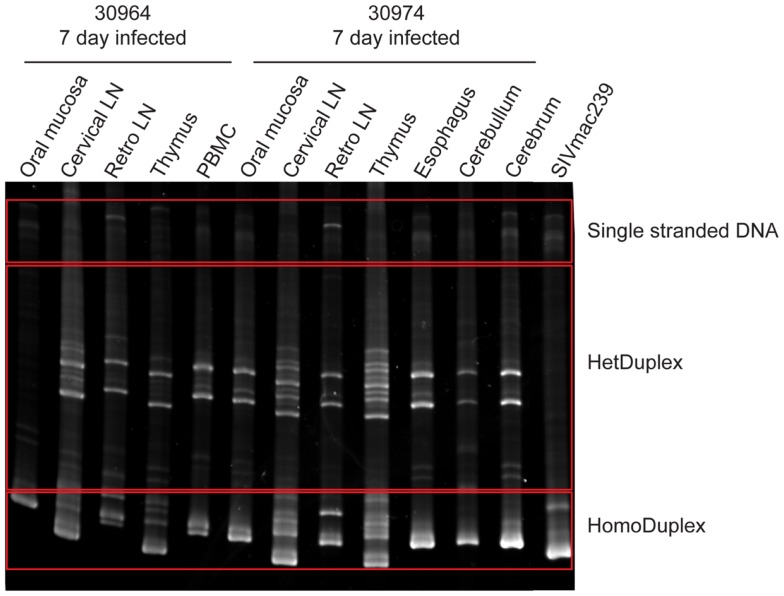
**Heteroduplex mobility assay of viral DNA from lymphoid, mucosal, and brain tissues**. The PCR amplified SIV envelope gene spanning the V1–V2 region was heated and rapidly re-annealed prior to separation by electrophoresis through a non-denaturing polyacrylamide gel and visualized by ethidium bromide staining. Two heteroduplex bands predominated in the majority of tissues examined indicating a limited viral diversity.

**Figure 3:**
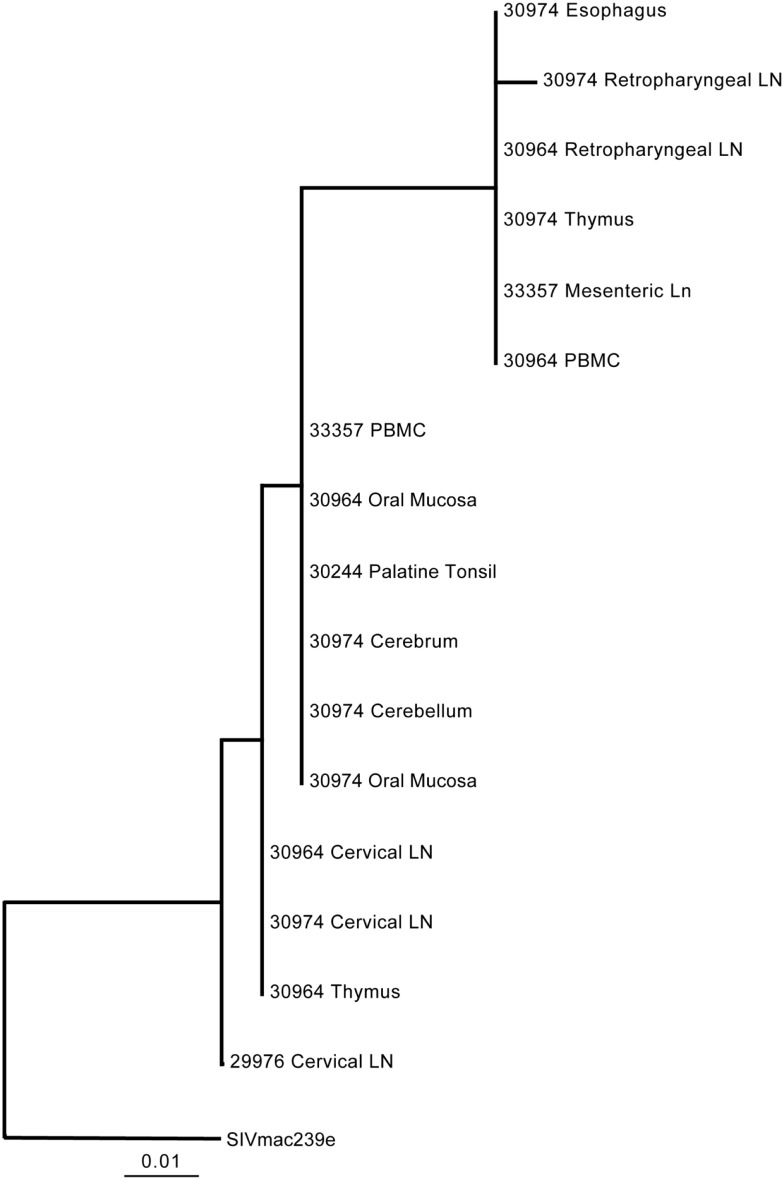
**Phylogenetic analysis of SIV Env V1–V2 regions in lymphoid and non-lymphoid tissues following oral inoculation**. The viral diversity within the first 7 days post-oral transmission of SIV results indicates the SIV *env* V1–V2 sequences in the cerebrum and cerebellum are closely related to sequences found in other lymphoid tissues.

Previous studies utilizing intravenous transmission have assessed whether tissue-specific viral sequestration occurs, and although sequestration can be observed in some studies, others have observed a more random distribution of variants in different tissues (18–20, 28). Here, the ability of the virus to gain access to the brain tissues within 1–2 days post-mucosal transmission raises the question as to whether the variants entering the brain at these early time points are distinct from those found in the periphery. To evaluate this question, a 590 bp DNA fragment encompassing the V1–V2 regions of SIV was PCR amplified from lymphoid and brain tissues and the diversity assessed by heteroduplex mobility assay (HMA) analysis. For DNA fragments of equal length, HMA requires a minimum of 1–2% mismatches in the sequences to detect heteroduplexes (29, 30). Utilizing HMA, two bands predominated in nearly all tissues assessed including the cerebrum and cerebellum indicating viral diversity was similar in all tissues assessed (Figure 2). To further support these findings, we performed sequence analysis of the 590 bp V1–V2 fragment. Neighbor joining phylogenetic analysis indicated viral sequences detected in cerebrum and cerebellum could also be found in other tissues (Figure 3). Taken together, these data suggest that once SIV crosses the mucosal barrier, the virus can spread quickly to all tissues of the body including the brain with no evidence for any tissue-specific viral sequestration.

## Discussion

Here we undertook a high dose oral administration of SIV to evaluate the rate at which SIV can be observed within CNS tissues following an oral infection. This oral infection route is relevant with regard to both mother-to-child transmission of HIV when virus is present within the breast milk (31) as well as oral-genital transmission when virus is present in semen (32). Our studies found SIV in brain tissues by 1–2 days post-inoculation indicating a rapid spread of the virus following oral transmission. We also determined that brain variants are similar to variants replicating in other tissues of the body and therefore no evidence for viral compartmentalization was observed in our study at these very early time points. It will be interesting to determine in future studies whether lower dose SIV mucosal inoculations at other mucosal sites result in a similar rate of spread throughout the lymphatics and into the CNS and whether this affects viral diversity in the different tissue compartments.

The use of saline perfusions to remove residual blood within the brain provides evidence that the SIV was present within the CNS tissue and not in the blood. Indeed, we were not able to detect SIV DNA in blood at 1 day post-infection and less than 50% of PCR reactions performed at 2 and 4 days post-infection were positive for SIV DNA. The extremely low levels of SIV DNA in the blood during the first 4 days post-infection and the observation that perfusion did not change the detection of SIV DNA in these tissues supports the conclusion that SIV DNA was within the CNS.

Our studies also tried to evaluate whether different tissue sections (i.e., cerebrum versus cerebellum) of the brain contained higher or lower amounts of SIV. Although our study did not observe a significant difference in the detection of SIV nucleic acid between the cerebrum and cerebellum, another study did find a preferential infection and intrinsic sensitivity to damage of particular areas of the brain using the SIV model (33). Furthermore, a detailed post-mortem assessment of HIV viral RNA levels in numerous areas of the brain indicated extensive intra- and inter-individual variability ranging from undetectable to greater than 10^7^ copies per gram of tissue (34). Our inability to observe any significant differences in the different tissues of the brain may be due to the early time points analyzed here in contrast to the more chronic samples evaluated in the other studies.

The limited viral diversity in the brain as well as other tissues of the body observed in this study indicates the bottleneck the oral/GI tract mucosal barrier imposes on the viral quasispecies. This is in agreement with previous studies demonstrating that vaginal transmission of SIV also results in lower viral diversity compared to IV inoculation (23, 35). Moreover, the rapid dissemination of SIV following oral inoculation is similar to the dissemination of SIV observed following vaginal inoculation (36). Indeed, as early as 1 day post-vaginal inoculation systemic lymphoid tissues as well as tissues of the intestinal tract have been found to contain viral RNA (36). Miller et al. did not however assess tissues of the CNS for viral presence at these very early time points (36). Interestingly, following oral or vaginal inoculation, viral RNA or DNA was not detectable in the plasma within the first 2 days post-inoculation suggesting the virus was trafficking to the different tissue compartments via the lymphatics.

The difficulties we faced to identify the cell types infected in these brains is likely due to a paucity of infected cells. However, using autologous dye-labeled lymphocytes Clay et al. have demonstrated that during acute SIV infection the elevated concentrations of CCL2 and CXCL9 in the CSF recruits monocytes from the blood into the choroid plexus of the brain (37). This may serve as a portal of entry for SIV, and likely HIV, during acute infection. However, it remains unknown as to whether these monocytes are productively infected or whether they are a conduit permitting infection of potential target cells in the brain.

The early penetration of virus into the CNS displayed here suggests that neural injury eventually resulting in HIV-associated neurocognitive complications and AIDS-associated dementia has the potential to begin shortly after infection. Indeed, one study in macaques demonstrated that neurocognitive changes can be measured within 3 months post-SIV infection and persist throughout infection (38). As the life expectancies among HIV-infected patients increase and as the population ages, neurocognitive impairments become a growing concern. Furthermore, current antiretroviral regimens do not appear to be able to effectively control viral replication in the CNS in all cases resulting in a potential source of residual virus in the CNS (16, 27, 39). This would suggest that early treatment with neuroprotective drugs might protect HIV-infected individuals from neurocognitive impairments or at least drastically slow this process down. Two very interesting neuroprotective molecules are the endogenous neurosteroids, dehydroepiandrosterone, and allopregnanolone, that not only have neuroprotective effects [reviewed in (40)], but also have antiviral properties (41–43).

There appears to be a general consensus that the infected cell type in the CNS is macrophage-like, although the exact subset(s) remains less clear [reviewed in (44)]. Estimates of the life span of perivascular macrophages, considered a target of HIV and SIV in the brain, range from days to weeks or longer according to one rhesus macaque study (45). The varying degrees of CNS penetrance of antiretrovirals (46) and the natural differences in turn-over of infected cells make it difficult to determine whether HIV can be completely eradicated from this tissue compartment. Early antiretroviral treatment, and possibly adjunctive treatments designed to specifically help the CNS, may help ameliorate CNS disease.

## Conflict of Interest Statement

The authors declare that the research was conducted in the absence of any commercial or financial relationships that could be construed as a potential conflict of interest.
